# Pervasive Differential Splicing in Marek’s Disease Virus Can Discriminate CVI-988 Vaccine Strain from RB-1B Very Virulent Strain in Chicken Embryonic Fibroblasts

**DOI:** 10.3390/v12030329

**Published:** 2020-03-18

**Authors:** Yashar Sadigh, Abdessamad Tahiri-Alaoui, Stephen Spatz, Venugopal Nair, Paolo Ribeca

**Affiliations:** 1 Avian Viral Oncogenesis, The Pirbright Institute, Ash Road, Woking GU24 0NF, UK; yashar.sadigh@pirbright.ac.uk; 2 Clinical BioManufacturing Facility, The Jenner Institute, University of Oxford, Old Road, Headington, Oxford OX3 7JT, UK; abdessamad.tahiri-alaoui@ndm.ox.ac.uk; 3 US National Poultry Research Center, 934 College Station Road, Athens, GA 30605, USA; stephen.spatz@ars.usda.gov; 4 Integrative Biology and Bioinformatics, The Pirbright Institute, Ash Road, Woking GU24 0NF, UK; 5 Biomathematics and Statistics Scotland (BioSS), James Clerk Maxwell Building, Peter Guthrie Tait Road, The King’s Buildings, Edinburgh EH9 3FD, UK

**Keywords:** Marek’s disease virus, transcriptomics, RNA splicing isoforms, MDV strain CVI-988, MDV strain RB-1B

## Abstract

Marek’s disease is a major scourge challenging poultry health worldwide. It is caused by the highly contagious Marek’s disease virus (MDV), an alphaherpesvirus. Here, we showed that, similar to other members of its *Herpesviridae* family, MDV also presents a complex landscape of splicing events, most of which are uncharacterised and/or not annotated. Quite strikingly, and although the biological relevance of this fact is unknown, we found that a number of viral splicing isoforms are strain-specific, despite the close sequence similarity of the strains considered: very virulent RB-1B and vaccine CVI-988. We validated our findings by devising an assay that discriminated infections caused by the two strains in chicken embryonic fibroblasts on the basis of the presence of some RNA species. To our knowledge, this study is the first to accomplish such a result, emphasizing how relevant a comprehensive picture of the viral transcriptome is to fully understand viral pathogenesis.

## 1. Introduction

Marek’s disease (MD) is a major scourge of poultry, caused by *Marek’s disease virus* (MDV, also known as *Gallid herpesvirus-*2, GaHV-2), a member of genus *Mardivirus* in the subfamily *Alphaherpesvirinae* of the family *Herpesviridae*. MD is characterised by paralysis, immunosuppression, and lymphoid infiltration into different tissues, including the peripheral nerves, eye, muscle, and skin. Lymphoid tumours in the visceral organs can be observed as early as 3 weeks post-infection. Control of MD has been achieved by vaccination with the live attenuated GaHV-2 strain CVI-988 (also known as Rispens) and the antigenically related non-oncogenic GaHV-3 vaccine strains such as SB-1, and *Meleagrid herpesvirus* 1 (*Herpesvirus of turkey*, HVT) strain Fc126 [1,2,3,4,5]. These vaccines were introduced at different periods to control the disease caused by various MDV pathotypes. Today, they are used individually or more frequently in combination because formulations, including more than one vaccine, can have a synergistic effect that induces stronger protection against MD [1]. However, despite the effectiveness of the single best vaccine (i.e., CVI-988) in producing lifelong immunity against clinical disease and mortality, all fail to produce sterilising immunity against MDV infection. Vaccinated chickens can become infected with virulent MDV (vMDV) field strains—they show no obvious clinical symptoms but shed virulent virus [6]. There has been a continuous evolution of MDV virulence, with the emergence of hypervirulent pathotypes [7,8]. A potential role of current vaccines at driving virulence is due to their inability to prevent infection and spread [6]. As vaccinated birds are still susceptible to superinfection by more virulent MDV subtypes, co-infection with vaccine and pathogenic strains are common in clinical materials such as poultry house dust and feathers [9].

A puzzling feature of MDV genomics is the very high sequence similarity between the virulent and vaccine strains. Despite the very different infection outcomes in vivo, the very virulent MDV strain RB-1B (vvMDV strain RB-1B) and the most effective MDV vaccine strain, CVI-988, differ by only about 1% of their sequences. In fact the genomic differences between the pathogenic RB-1B and cell culture adapted CVI-988 strains are so limited that only one DNA-based assay [10,11] is available to differentiate between them; the assay relies upon the detection of a few nucleotide substitutions localized to a single locus. Consequently, it would look like the differences at the DNA level are not the best proxy to understand why the two strains behave so differently.

A much better vantage point might be represented by the viral transcriptome—for one thing, it has already been shown in the past that several herpesviruses have an unsuspectedly rich repertoire of RNAs [12,13,14,15]. Detailed knowledge of the viral transcriptomes can also potentially shed more light on the different mechanisms taking place during virus–host interaction. In this study, we sought to establish a way of distinguishing pathogenic MDV from the CVI-988 vaccine strain by the detection of RNA transcripts differentially expressed by either strain in chicken embryo fibroblast (CEF) cells. This led to the discovery of pervasive strain-dependent splicing, with markedly different splicing isoforms (spliceforms) expressed in the infected cells despite the fact that the two strains are closely related. Alternative splicing plays an important role in many biological processes, and its deregulation in humans can result in a number of congenital diseases [16]. In cancer cells, alternative spliceforms are reported for many proteins, and some of them used as biomarkers in diagnostics [17,18,19].

In the work presented here, we report novel differential splicing patterns between the pathogenic (RB-1B) and non-pathogenic (CVI-988) strains of MDV. To the best of our knowledge, this is the first time that differential splicing in two closely related strains of viruses is reported. Following its discovery using bioinformatic approaches, we directly validated it in vitro, using techniques based on real-time PCR. 

## 2. Materials and Methods

### 2.1. RNA-Sequencing

#### 2.1.1. Cell and Virus History

Primary CEF cells were prepared from 10-day-old Valo or line 0 embryos. All of the viruses were passaged on primary CEF cells. CEF cells that were prepared from line 0 were used to generate RNA-Seq data, and CEF cells that were prepared from Valo eggs were used for validation experiments.

Very virulent MDV strain RB-1B was obtained from the splenocytes of a bird (wing band no. 3345/KV16), which was infected with vvMDV strain RB-1B (no. 3345/KV16) and demonstrated clinical signs. Briefly, the splenocytes were used to infected CEF cells to prepare a first passage of the RB-1B virus stock. A second passage of MDV strain RB-1B was prepared using the first stock and used for RNA isolation, as described in the next section. MDV strain CV-I988 was prepared from CEF cells with two passage history after they were infected with Nobilis Rismavac vaccine virus. The passage two virus stock was used for RNA isolation as the cell culture adapted the virus strain, as described below.

#### 2.1.2. Sample Preparation

Confluent CEF cells in a 75 cm^2^ flask (8.0 × 10^6^ cells in each flask) were infected with 1500 pfu of CVI-988 or RB-1B virus in Dulbecco’s modified Eagle medium (DMEM) containing 5% FBS and the antibiotics streptomycin (100 μg/mL) and penicillin (100 U/mL). 

The flasks were incubated for 5 days at 37 °C in 5% CO_2_, and RNA was isolated using Trizol (Thermofisher Scientific, Waltham, MA, USA) according to the method described by the manufacturer. For each biological condition (CEF cells infected with vvMDV strain RB-1B; and CEF cells infected with MDV CVI-988 vaccine strain), two biological replicates were selected.

Feather samples from commercially kept vaccinated chicken were obtained from sample archive of Zoetis, France. For vvMDV strain RB-1B-positive samples, feathers samples were obtained from samples that were kept in the samples archive of the avian viral oncogenesis group (AVO) at The Pirbright Institute during past years. 

#### 2.1.3. Sequencing

RNA samples were sequenced at the Centro Nacional de Análisis Genómico (Barcelona, Spain). Briefly, total RNA was assayed for quantity and quality using Qubit RNA HS Assay (Thermofisher Scientific, Waltham, MA, USA) and RNA 6000 Nano Assay on a Bioanalyzer 2100. The experimental protocol to construct stranded mRNA RNASeq libraries starting from the total RNA employed the TruSeqStranded mRNA LT Sample Prep Kit (Illumina Inc., Rev.E, October 2013, San Diego, CA, USA). The initial input was 0.5 ug of total RNA for each sample. The size and quality of each final library were validated on an Agilent 2100 Bioanalyzer with the DNA 7500 assay (Agilent, Santa Clara, CA, USA). Libraries were sequenced using TruSeq SBS Kit v3-HS in paired-end mode with read length 2 × 76 bp. Each sample was sequenced in a fraction of a sequencing lane on a HiSeq2000/2500 machine (Illumina, San Diego, CA, USA) following the manufacturer’s protocol, generating between 59 and 87 million paired-end reads per sample. Images analysis, base calling, and quality scoring of the run were performed using the manufacturer’s software Real-Time Analysis (RTA 1.13.48) and were followed by generation of FASTQ sequence files with CASAVA 1.8. For this analysis, the two biological replicates available for each MDV strain (RB-1B and CVI-988) were pooled together before analysis, to increase the overall read coverage. 

### 2.2. Bioinformatics Selection of Biomarkers

#### 2.2.1. Primary Analysis

**Figure 1:**
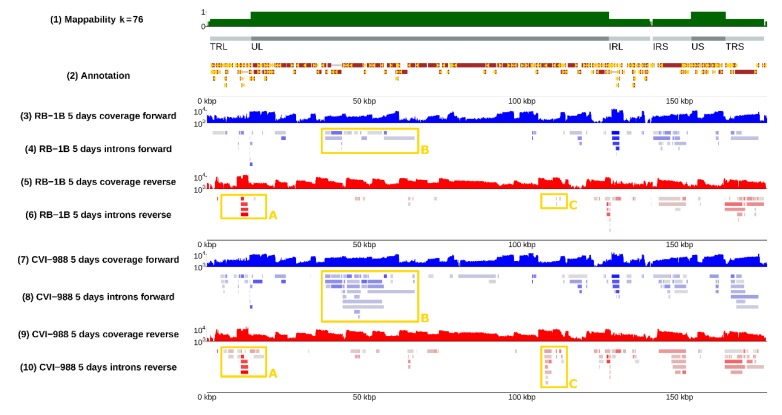
The splicing landscape of Marek’s disease virus (MDV)-infected chicken embryo fibroblast (CEF) cells. An overview of splicing in CEF cells infected with two strains of MDV at 5 days post-infection. Results are presented as a genome browser, each numbered label on the left corresponding to a different track on the right. Track (1) (green) shows mappability—computed as in [22]—for all 76-mers in the MDV genome, which matched the read length of our RNA-sequencing experiment. Areas having mappability 1 corresponded to unique regions, whereas areas having mappability 0.5 corresponded to parts of the genome repeated twice. Track (2) (brown) shows the position of the open reading frames originally annotated by Tulman and colleagues [37,38,39] plus a few additional transcripts published more recently; gene nomenclature was omitted to reduce clutter. Blue profile tracks (3) and (7) show the coverage of directional RNA-Seq along the forward strand in different biological conditions – infection with MDV strain RB-1B for track (3), and infection with MDV strain CVI-988 for track (7). Red tracks (5) and (9) show the corresponding coverage along the reverse strand of the virus. Tracks (4), (6), (8), and (10) show observed introns, as deduced from coverage in spliced reads. The degree of coverage of an intron corresponds to how coloured it is with respect to its grey background. Introns tracks are also directional (forward and reverse), and to each coverage track, there corresponds an intron track (see labels). Placement of an intron in the inverted repeats – terminal repeat long (TRL) vs. internal repeat long (IRL) or internal repeat short (IRS) vs. terminal repeat short (TRS) – was done arbitrarily. There are several regions of the genome showing substantially different splicing patterns for the RB-1B and CVI-988 strains. Splice junctions of interest within such regions are highlighted with yellow frames. Frame A is magnified in Figure 2; frame B in Figure 3; frame C in Figure 4. This figure can be reproduced in the online MDV genome browser by accessing https://mallorn.pirbright.ac.uk/browsers/MDV-annotation/Figure-1.html.

Reads were subjected to preliminary quality control and processed with a pipeline for primary data analysis based on the GEM mapper [20], which is an evolution of the one used to process the data produced by the GEUVADIS consortium [21]. Contrary to other modern methods relying on accurate annotations, this pipeline includes a highly sensitive de novo intron detection step. This allows for accurate alignments despite errors or limitations in the available annotation of cellular transcripts and enabled an unbiased picture of splicing in different MDV strains. We point out that separately aligning MDV RB-1B reads to the RB-1B genome and MDV CVI-988 reads to CVI-988 would not have been possible for this analysis, as during subsequent stages we needed to compare coverage of the same intron across different viruses. To make results comparable across conditions, reads from samples infected with different viruses (MDV RB-1B and CVI-988) were all aligned to the same MDV MD5 reference (NCBI accession number AF243438). Although in principle this procedure might decrease the number of reads successfully mapped and potentially introduce artefacts, in practice it works well due to the high sequence similarity of the strains involved in the experiment; the conclusions presented in the paper are qualitatively identical when reads from both infections (RB-1B and CVI-988) are aligned to either RB-1B (NCBI accession EF523390) or CVI-988 strain (accession DQ530348). As a relevant fraction of the MDV genome was replicated twice (see, for instance, Figure 1, where mappability [22] for the genome is shown), reads aligning to more than one location of the genome were assigned to all locations, with normalisation 1/(number of alignments). Keeping only uniquely mapping reads, as is often done in RNA-Seq data analysis, would have resulted in complete loss of signal in the repeated regions.

#### 2.2.2. Tentative Annotation of Full Spliced Coding Transcripts

The pipeline used for primary analysis of each sample generated a list of introns, each one annotated with the number of spliced reads covering it. Introns having a coverage of 10 reads of more were kept, candidate exons were deduced from them, and an in-house script was used to generate all possible coding sequences of exons compatible with translation start and end signals present in MDV genome. Only putative transcripts leading to protein sequences longer than 35 amino acids were kept. This step was performed separately for the RB-1B and the CVI-988 data.

#### 2.2.3. Selection of Relevant MDV Encoded Introns

The intron list obtained after primary analysis was split on the basis of the following criteria: (I) viral introns that had a sufficient read coverage in CEF cells infected with vvMDV strain RB-1B with no coverage in CEF cells infected with CVI-988 vaccine, (II) viral introns having sufficient read coverage in CEF cells infected with attenuated CVI-988 with no coverage in CEF cells infected with RB-1B. It should be pointed out that having zero RNA-sequencing reads for a feature in one condition did not mean that that feature was not expressed in that condition—it simply means that the expression level of the feature was too small for the dynamic range of the experiment, which was determined by the expression level of the most abundant feature and the number of sequencing reads produced during the experiment. Introns were considered to be sufficiently populated whenever they had non-zero coverage in all biological replicates, and the sum of their coverage across all replicates was ≥20. Finally, the list of introns was prioritised on the basis of the geometric mean of the coverage in spliced reads across all replicates. An arbitrary final minimum threshold of 25 was used for the mean, in order to exclude from the list candidates with low-level expression.

#### 2.2.4. Differential Expression of Transcripts

The differential expression for ICP27 mentioned in the Discussion section was computed using edgeR [23] version 3.20.9.

#### 2.2.5. Data Visualisation

The genome browser is based on a customised version of JBrowse 1.11.6 [24].

### 2.3. Real-Time PCR Validation of Viral Junctions

#### 2.3.1. Sample Preparation

MDV strains RB-1B and CVI-988 were propagated as previously described [10,11]. To prepare samples for real-time PCR, CEF cells were infected in 9.8 cm^2^ wells (6-well plates, 1.3 × 10^6^ cells in each well) with 500 pfu of MDV strains RB-1B or CVI-988. Uninfected and infected cells were harvested for RNA purification using Trizol reagent (Thermofisher Scientific, Walthan, MA, USA), as described by the manufacturer. 

For the time-course experiment, semi-confluent CEF cells (80%) were transfected with 1 μg of RB-1B or CVI-988 bacterial artificial chromosome (BAC) DNA clone using 10 μL of lipofectamine transfection reagent (Life Technologies) in 9.8 cm^2^ tissue culture plates. Three independent transfections were conducted to have three replicates (*N* = 3). At 6, 12, 18, 30, 42, 54, 66, 87, and 90 h post-transfection, CEF cells were harvested from the plates, washed with PBS, suspended in RLT buffer (Qiagen, Hilden, Germany), and stored at −80 °C until the time of RNA purification. RNA was isolated using RNeasy (Qiagen), and the contaminating DNA was destroyed by DNaseI treatment (New England Biolabs, Ipswich, MA, USA). Complementary DNA (cDNA) was synthesized using RevertAid H minus reverse transcriptase (ThermoFisher Scientific) with random hexamers. Real-time PCR for I1 and *Glyceraldehyde 3-phosphate dehydrogenase* (*GAPDH*) was conducted as described in [11,25]. 

RNA from feather samples was obtained using RNeasy Mini (Qiagen) kit. Briefly, pulps from two or three feathers were cut and sliced into small pieces and were further homogenised inside the RLT buffer provided by the manufacturer using 1 mm glass beads in a Biospec Minibeadbeater device. The homogenised lysate was loaded onto a Qiagen RNeasy column. The RNA sample was used immediately to synthesise cDNA from. The cDNA was synthesized using RevertAid H minus reverse transcriptase (ThermoFisher Scientific) using the specific reverse primer for I1 (Table S3). 

To calculate gene expression between the two strains of MDV, the delta delta CT (ΔΔ*C*T) approach was used [26]. Δ*C*T for MDV strain CVI-988 and MDV strain RB-1B were normalised against the *GAPDH* housekeeping gene. The difference between the Δ*C*T values of the two strains was calculated as ΔΔ*C*T. Alternatively, in a second series of measurements the viral reference (V.Ref) gene, as defined in Table 3, was used as the housekeeping gene.

#### 2.3.2. Primers and Probes

Primers and probes were designed for each splicing isoforms using the PrimerQuest tool provided by Integrated DNA Technologies (IDT). Each probe was designed to span the splice junction to avoid non-specific interaction between closely related splice variants. A list of primers and probes, sizes of the amplicons, and locations of targeted introns are provided in Table S3. *GAPDH* was used as the host gene. A pair of primers and a probe for the splice junction between exons 5 and 6 was designed to be used to detect the level of *GAPDH* cDNA. Each probe was labelled with 5′FAM reporter, ZEN, and 3′-BHQ1 quenchers (Integrated DNA Technology, IDT). The sequences for primers and probes are provided in Table S3.

#### 2.3.3. Relative Quantitative RT-PCR

qRT-PCR was performed using ABsolute blue QPCR mix (Thermo Scientific), primer pairs (each 0.4 μM), and probe (0.2 μM) for the splice junctions of interest. To generate a standard curve, splice junction amplicons were cloned in pGEM-T plasmid (Promega, Madison, WI, USA) and subjected to Sanger sequencing. Tenfold serial dilutions were prepared to produce a range from 1 nM to 10 fM. Real-time PCR reactions were run in triplicates, with individual reactions for the splice junctions, *GAPDH* and the virus controls. The reactions were processed on a 7500 Fast Real-time PCR system (Applied Biosystems, Foster City, CA, USA) with reaction conditions specified by the master mix manufacturer. Data were collected and analysed using 7500 Software (v2.3, Applied Biosystems). Template DNA was replaced with its RNA predecessor in the no template control samples, except for the samples derived from the feather follicles, with which the cDNA was replaced with distilled water. 

#### 2.3.4. Accession Numbers

The raw sequencing reads were deposited into the NCBI Sequencing Read Archive (SRA) under the project accession number PRJNA541962. The files corresponding to CEF cells infected with RB-1B and CVI-988 strains, each in two replicates, can be downloaded as accession numbers SRR9030404, SRR9030405, SRR9030406, and SRR9030407.

## 3. Results

Briefly, we infected chicken embryo fibroblast (CEF cells) with two strains of MDV: vvMDV strain RB-1B and the vaccine strain CVI-988. RNA was extracted from the cells, and a polyA-enriched fraction was used to prepare complementary DNA (cDNA). cDNA was sequenced using Illumina technology (HiSeq 2000/2500) with a directional protocol. Reads were mapped to several MDV strains using a sensitive analysis pipeline (see the **Materials and Methods section** for more details).

The number of reads mapped with high alignment scores to the genome of CVI-988 or RB-1B, as determined by the analysis pipeline, are shown in Table 1. Raw reads were deposited into the NCBI Sequence Read Archive under project accession number PRJNA541962.

### 3.1. Splicing Was Pervasive in MDV

In CEF cells, the viral transcriptome of the MDV strains we tested consisted of a complex landscape of splicing isoforms, most of which were novel and have not been reported or included in the official genome annotations. This is similar to RNA-sequencing results previously published thus far for all subfamilies of herpesviruses: the alphaherpesviruses *Herpes simplex virus 1* (HSV-1) [12,13] and *Pseudorabies virus* (PRV) [27,28], the betaherpesvirus *Cytomegalovirus* (CMV) [14], and the gammaherpesvirus *Epstein-Barr virus* (EBV) and *Kaposi’s sarcoma associated herpes virus* (KSHV) [15]. In particular, comparable complexity was observed with CMV [14] or EBV [29] viruses. In CMV infected cells, a total of 751 ORF were identified, which are transcribed and translated into polypeptides into proteins [14]. During EBV reactivation, a high level of complex bidirectional transcription was observed [29]. Sequencing methods used in the literature include Illumina, Pacific Biosciences, and Oxford Nanopore technologies.

In detail, our analysis of spliced sequencing reads (see the **Materials and Methods section**) revealed 154 introns in MDV strain RB-1B and 246 in MDV strain CVI-988 after filtering out introns with low coverage (<10 reads) and introns that were not spliced in all biological replicates (the complete list can be found in Table S1). A small proportion of these introns, specifically those in the spliceforms of *Meq*, *vIL18*, *ICP4*, *gC*, *pp38*, *MDV012*, and *latency associated transcript* (*LAT*) had already been identified and annotated [30,31,32,33,34,35,36], and were affirmed by this study. The localization of the introns on the MDV genome is presented in Figure 1 (see tracks 4, 6, 8, and 10), where each solid bar represents a different intron. The direction of splicing at acceptor/donor sites is indicated with a yellow arrow. 

**Figure 2:**
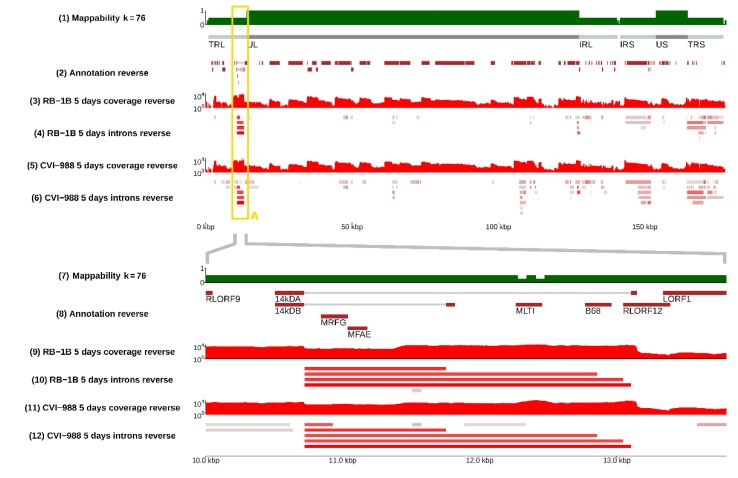
The splicing landscape at the junction between terminal repeat regions. **Top panel**: negative strand of the whole MDV genome; **bottom panel**: region from 10 to 14 kb (TRL/UL junction). The **bottom panel** is a magnified version of the content of the yellow frame A present in the **top panel** and in Figure 1. Alternative spliceforms across the 14KD polypeptide (*pp14*) gene of MDV in strains RB-1B and CVI-988 are compared. From the RNA-sequencing signal, one can see four main alternative spliceforms in CEF cells infected by RB-1B, whereas five alternative spliceforms were identified in cells infected by CVI-988. As shown in track (8) (annotation), only two isoforms (14 kDA and 14 kDB) were present in the standard MDV annotations, as defined by Hong and Coussens [40]. The **bottom panel** of this figure can be reproduced in the online MDV genome browser by accessing https://mallorn.pirbright.ac.uk/browsers/MDV-annotation/Figure-2.html.

**Figure 3:**
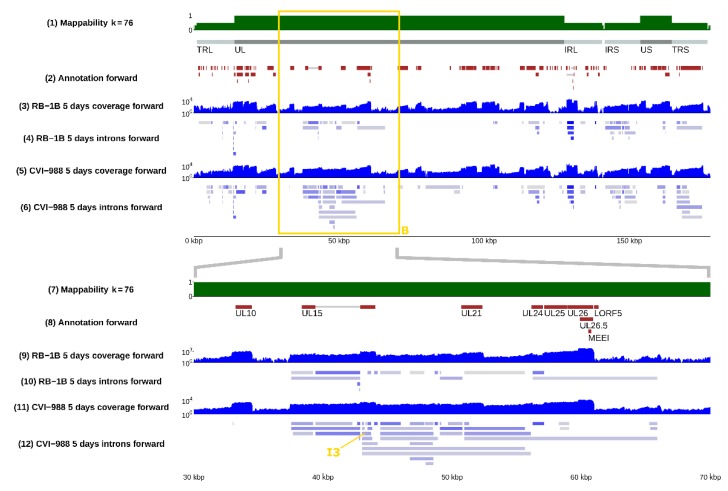
The landscape of alternative splicing in the unique long region of MDV. **Top panel**: positive strand of the whole MDV genome; **bottom panel**: the region between positions 30 and 70 kb. The **bottom panel** is a magnified version of the content of the yellow frame B present in the **top panel** and in Figure 1. Although many annotated and unannotated introns were present along this area in RB-1B during its infection of CEF cells – track (9) – the complexity of the splicing landscape in the same region was vastly superior in CVI-988, which showed several times more introns being actively spliced – track (11). Intron I3, which is one of the several expressed in CVI-988 and not in RB-1B, is highlighted with a yellow arrow in the **bottom panel**. The **bottom panel** of this figure can be reproduced in the online MDV genome browser by accessing https://mallorn.pirbright.ac.uk/browsers/MDV-annotation/Figure-3.html.

Several positions appeared to be alternative splice donors/acceptors, with several possible corresponding introns being selected by the splicing machinery (see Figure 2). The likelihood of such choice, which can be evaluated from RNA-sequencing data by comparing the coverage of the different splice junctions, is highly variable—it can be very similar or very different depending on the specific splice site. The distribution of splicing donors/acceptors was found to also not be uniform across the whole genome, but rather concentrated in some specific regions, especially the inverted repeats and, surprisingly, the unique long region between 30 and 70 kb (see Figure 1 and Figure 3).

An interactive version of the data, presented as a genome browser, can be publicly accessed at https://mallorn.pirbright.ac.uk/browsers/MDV-annotation/.

### 3.2. Splicing Was Strain-Specific, and Occurred More Frequently in CVI-988

**Figure 4:**
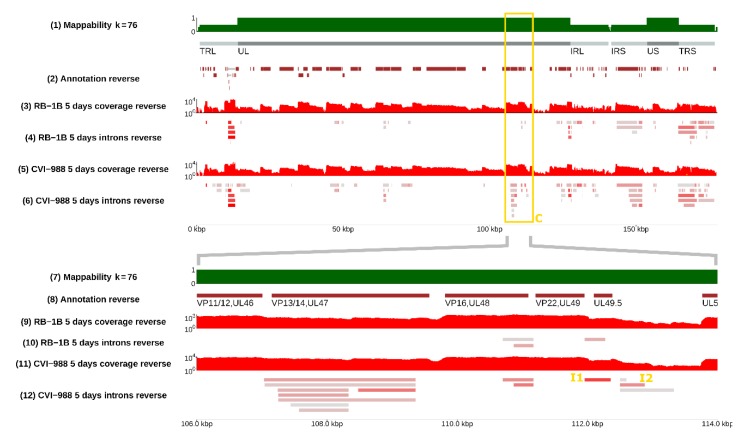
More splicing events on the reverse strand of MDV. **Top panel**: negative strand of the whole MDV genome; **bottom panel**: the region between 106 and 114 kbp. The **bottom panel** is a magnified version of the content of the yellow frame C present in the **top panel** and in Figure 1. In this region, more splicing events were observed in MDV strain CVI-988 during its infection of CEF cells compared to MDV strain RB-1B. In particular, one of the unique introns (named I1 in Table 3), identified in MDV strain CVI-988 between the *UL49.5* and *UL49* genes, was detected with a high read coverage (613 spliced reads), whereas in RB-1B the same isoform does not exist and a shorter intron can be observed. Introns I1 an I2 are also highlighted in yellow in the **bottom panel**. The **bottom panel** of this figure can be reproduced in the online MDV genome browser by accessing https://mallorn.pirbright.ac.uk/browsers/MDV-annotation/Figure-4.html.

Many splicing isoforms appeared to be produced by both strains, as expected, given their similarity in nucleotide space. Despite this, one of our major findings was the identification of specific genomic regions that encode a number of isoforms, which were found to be strain-specific, at least in infected CEF cells. Examples are illustrated in Figure 2, where the presence of different spliceforms within the 14 kD locus is reported; in Figure 3, showing how a much greater number of spliced introns is encoded in the CVI-988 genome relative to those in the RB-1B genome, specifically on the negative strand of the UL region (coordinates 10–70 Kb); and in Figure 4, which reveals that this was not restricted only to the negative strand. Many similar examples of varying splicing isoforms could be found throughout the MDV genomes, corroborating the observation that the splicing patterns of RB-1B and CVI-988 are significantly different, with much higher levels of splicing occurring in CVI-988-infected CEF cells.

### 3.3. Tentative Reannotation of Coding Transcripts

In genomic regions with many consecutive multiple splicing donors/acceptors, such as the one shown in Figure 3, the combinatorics of alternative splicing are too complex to be solved with short reads. Further complementary investigations based on long-read technologies (e.g., Pacific Biosciences) will be required to fully resolve the genomic structure of long spliced transcripts being made of many consecutive exons. However, it is possible to exhaustively enumerate all coding spliced transcripts compatible with the introns observed. Here, we took such an approach to map sequences of putative exons to all open reading frames, which would translate into a protein longer than 35 amino acids (see the Materials and Methods section). It should be emphasised that some of such predicted coding transcripts (in particular the more complex ones involving more than two exons) may not have been present at all, and confirming their presence experimentally is outside of the scope of this manuscript. However, this tentative reannotation gives a good idea of the splicing complexity at different viral loci. One should also point out that our tentative reannotation only considered coding transcripts, but many more non-coding viral transcripts might potentially be found as well. 

**Figure 5:**
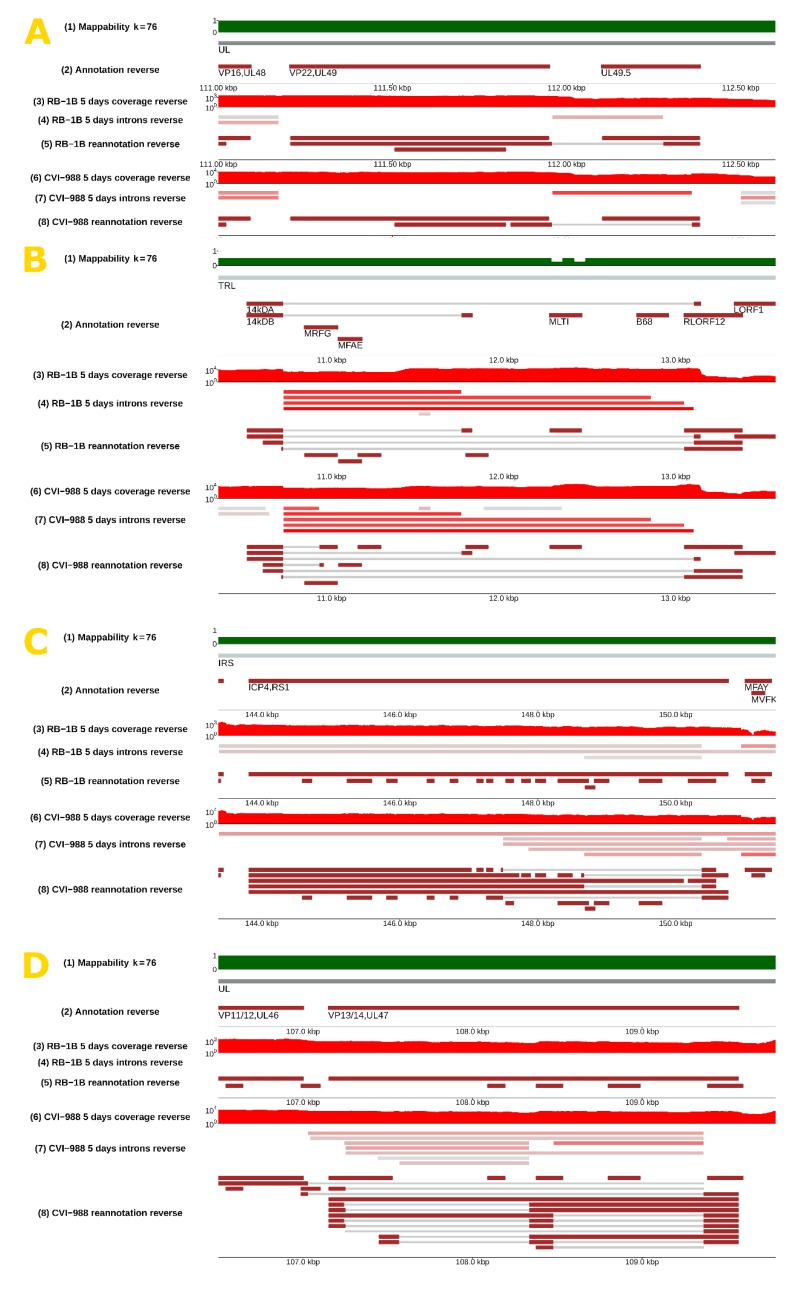
Tentative condition-dependent reannotation of splicing events on the reverse strand of MDV. In silico reconstructed coding spliced transcripts compatible with the introns observed from Illumina short sequencing read—the coding spliceforms shown in each panel were produced by automatic prediction methods (see the **Materials and Methods section**), but the presence of full-length transcripts was not experimentally validated. We show the likely presence of new spliceforms for genes *UL49.5/UL49* (**panel A**), *ICP4* (**panel B**), *PP14* (**panel C**), and *VP13/14/UL47* (**panel D**). The panels of this figure can be reproduced in the MDV genome browser by accessing **Panel A**: https://mallorn.pirbright.ac.uk/browsers/MDV-annotation/Figure-5A.html **Panel B**: https://mallorn.pirbright.ac.uk/browsers/MDV-annotation/Figure-5B.html **Panel C**: https://mallorn.pirbright.ac.uk/browsers/MDV-annotation/Figure-5C.html **Panel D**: https://mallorn.pirbright.ac.uk/browsers/MDV-annotation/Figure-5D.html.

The reannotations can be accessed and downloaded using the feature “Save track data” through the genome browser mentioned above. Table 2 lists the number of putative coding spliceforms annotated for many relevant MDV genes when CEF cells were infected by either RB-1B or CVI-988 MDV strains. In Figure 5, we illustrate four cases in more detail. In panel A, we show splicing events occurring in *UL49*/*UL49.5* after infection by RB-1B or CVI-988 (the latter event is referred to as I1 in Table 3). Each splicing event was unique, and the nucleotide sequences of the two spliceforms differed. In addition, I1, the spliceform specific to CVI-988, was present with higher read coverage (Table 3). Panel B describes six new possible splicing isoforms in the *ICP4* gene of MDV strain CVI-988. Additionally, we observed more splicing events that could not be assigned to the *ICP4* gene itself as they were detected on the opposite strand. Panel C shows two new putative splicing isoforms for *pp14* occurring in both MDV strains (in addition to the two already known) and another two novel isoforms occurring only in CVI-988. Panel D displays 14 novel splicing events occurring at the *UL52*/*UL53*/*UL54* loci in CVI-988.

Our data confirms many MDV spliceforms previously reported in the literature. The splicing variant, which was reported [34] between the *ORF011* and *ORF012* genes of MDV, can be found in our RNASeq data—it was annotated on the genome browser as RB-1B-0865 or CVI-988-0883. In addition, we observed both spliced variants of *pp38* in CEF cells infected with MDV strain RB-1B (RB-1B-0918 and RB-1B-0919, according to the nomenclature used in our reannotation) and one spliced variant of *pp38* in cells infected with CVI-988 (CVI-988-0979). In the *gC* region, we observed the two previously described splicing transcripts [33], namely, RB-1B-0874 and RB-1B-0875; or CVI-988-0920 and CVI-988-0921 in RB-1B- and CVI-988-infected CEF cells, respectively. Additionally, we observed three further novel spliced isoforms: RB-1B-0933, RB-1B-0876, and RB-1B-0877 in CEF cells infected with the RB-1B strain, as well as CVI-988-1051, CVI-988-0922, and CVI-988-0923 in CEFs infected with strain CVI-988. We observe a higher diversity in transcripts of the ICP4 gene in cells infected with MDV strain CVI-988 than in cells infected with MDV strain RB-1B.

Although our data confirm many known spliceforms of MDV, we could not identify all of them. For example, we could not observe the spliceform between the *Meq* and *IL8* loci, which was described as *meqΔC-BamL* in MSB-1 cells or infected CEF cells [30,41]. Unlike other previous reports [31], we only observed one unspliced product for the Meq protein. In the *IL8* region, we did not observe *RLORF4/IL8*- or *RLORF5/vIL8*-related transcripts [31]. According to our data, an alternative start codon in the *vIL18* region could be employed to produce a novel transcript for *vIL8* made of exons I and II of the gene, but representing an overlapping ORF (RB-1B-0928 or CVI-988-1019) to the main ORF of *vIL8* (RB-1B-0936 or CVI-988-1068). In the *vIL8* region, splicing between exon I and exon II would produce a shorter transcript than the full *vIL8* transcript, with a length of 379 nucleotides and containing an immature stop codon at the end of the second splice site (RB-1B-0929 or CVI-988-1020). We examine possible reasons for these discrepancies in the Discussion section.

### 3.4. Selection of Strain-Dependent Introns

By comparing the coverage in spliced reads at the same genomic positions between the vvMDV strain RB-1B and vaccine strain CVI-988, a list of viral introns showing the highest variation between strains was generated (Table 3, see the **Materials and Methods section** for a description of the procedure used). According to this coverage-based ranking and feasibility of real-time PCR probe design, three introns that were exclusively spliced in the CVI-988 transcriptome (indicated in Table 3 as I1, I2, and I3) were selected for further study. Introns I1 and I2 are highlighted in Figure 4, whereas intron I3 can be found in Figure 3. A viral junction (V.Ref) was selected on the basis of our RNA-sequencing data and equal expression levels in CEF cells infected with MDV strain RB-1B or MDV strain CV-I988.

### 3.5. The Kinetics of I1 Expression in CEF Cells Transfected with CVI-988 and RB-1B

**Figure 6:**
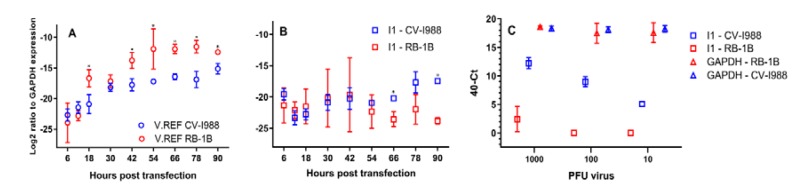
**Panel A**. Expression level of V.Ref in CEF cells transfected with bacterial artificial chromosome (BAC) DNA. Level of V.Ref was measured in cells harvested at time points 6, 12, 18, 30, 42, 54, 66, 78, and 90 h post-transfection. The expression levels of V.Ref were calculated and normalised to the corresponding levels for *GAPDH* and the ratios displayed on the graph for each time point. Three independent biological replicates were measured per time point. *P*-values were computed with a multiple *t*-test. *P*-values for the different time points were 0.5, 0.15, 0.04, 0.22, 0.03, 0.06, 0.007, 0.015, and 0.015. **Panel B**. Expression of I1 in CEF cells transfected with RB-1B or CVI-988 BAC DNA as a function of time. Cells were harvested at time points 6, 12, 18, 30, 42, 54, 66, 78, and 90 h post-transfection. Expression levels of I1 were calculated and normalised to the corresponding levels for *GAPDH* (see the **Materials and Methods section**), and the ratios are displayed on the graph for each time point. Three independent biological replicates were measured per time point. *P*-values were 0.37, 0.29, 0.50, 0.81, 0.88, 0.41, 0.008, 0.06, and 0.00067. *P*-values were computed with a multiple *t*-test. **Panel C**. Expression level of *GAPDH* and I1 at 5 days post-infection in CEF cells infected with 1000, 100, or 10 PFU of MDV strains CVI-988 or RB-1B. Three independent biological replicates were measured per PFU value. Note that the *x*-scale is not continuous—there were only three discrete values corresponding to 1000, 100, and 10 PFU. In **panels A and B**, asterisks (*) indicate statistically significant data points.

The expression level of I1 was determined using qRT-PCR using RNA isolated from CEF cells infected with both RB-1B and CVI-988. MDV is avidly cell-associated, which makes it problematic to achieve synchronised expression of viral transcripts due to in vitro growth differences between the vvMDV strain RB-1B and strain CVI-988. Hence, a time-course experiment was performed by transfecting CEF cells with equimolar amounts of infectious bacterial artificial chromosome (BAC) clones of CVI-988 and RB-1B [42,43] (**Materials and Methods**). Expression levels for I1 and virus reference (V.Ref) introns were calculated from transfected cells harvested at 0, 6, 12, 18, 30, 42, 54, 66, 78, and 90 h post-transfection, using *GAPDH* as a reference for relative quantitation (Figure 6 panels A and B). 

According to our RNA-Seq data, V.Ref was expressed at the same level in CEF cells 5 days post-infection. However, in the infectious BAC DNA transfection assay, we would expect to see similar expression levels for CVI-988 or RB-1B in the transfected cells. However, a higher expression of V.Ref in cells that were transfected with MDV strain RB-1B BAC DNA clone was observed (Figure 6, panel A). The expression level of V.Ref in the two transfected cell lines became very close at 90 h post-transfection, but a statistically significant difference remained between the two.

Intron I1 was expressed at roughly equal levels in both RB-1B and CVI-988 infected cells until 54 h post-transfection (Figure 6 panel B), the baseline being that *GAPDH* was about 4 × 10^6^ times more expressed than I1. However, starting from 66 h post-transfection, the expression of I1 progressively increased (with differences becoming statistically significant) in cells transfected with CVI-988. The peak was recorded at 90 h post-transfection, when the expression level was about 80 times greater in cells transfected with CVI-988 than in cells transfected with RB-1B. This finding agrees with the earlier observation deduced from RNA-sequencing data that at 3 days post-infection, and increasingly so, I1 is expressed at exceedingly greater levels in CEF cells infected by CVI-988 than in cells infected by RB-1B.

### 3.6. I1 Could Be Detected at a Very Low Level

The expression differentials previously described remained true at 5 days post-infection across a wide range of infectious virus loads. The expression levels of I1 were quantified using relative RT-PCR with RNA isolated from 1.3 × 10^6^ CEF cells infected with 1000, 100, or 10 PFU of MDV strains RB-1B or CVI-988 in 9 cm^2^ tissue culture dishes (Figure 6 panel C). Due to the cell-associated nature of MDV, we were unable to calculate multiplicity of infection (MOI). The following list contains the results:

1. The expression of the housekeeping gene *GAPDH* remained unchanged at a 40-*C*t value of approximately 18 across the different PFU levels (*p* = 0.48 on the basis of a two-way repeated measures ANOVA test) and independent of the strain.
2. In CEF cells infected by CVI-988, the levels of expression of I1 correlated very well with inoculum titres (Pearson’s *r* = 0.98, *p*-value = 6.1 × 10^−6^), and I1 was detectable across the whole PFU range.
3. I1 expression was undetectable in 1.3 × 10^6^ CEF cells infected with 100 or 10 PFU of RB-1B. In cells infected with 1000 PFU, I1 levels were still extremely low (averaged 40-*C*t value of 2.4).

Overall, our results support the use of intron I1 as a good candidate strain-specific spliceform across a wide range of viral titres. Whenever it should be detectable, i.e., in CEF cells infected by CVI-988 starting from roughly 60 h post-infection, it remained so even at a very low virus input, and its abundance at 5 days post-infection in vitro showed a very clear and predictable linear relationship with virus input. We assumed that these findings would be generalizable to I2 and I3.

To evaluate the presence of I1 in the infected tissue/clinical samples, the real-time PCR assay was conducted on cDNA, which was made from RNA isolated from feather pulps. Figure S1 shows the result of the real-time assay. As it is shown in Figure S1 panel A, three out of four samples, which were tested for the presence of I1 in the vaccinated group, produced a positive signal during the PCR reaction (40-*C*t = 7.14, 3, and 4.7 for CVI-988 1, CVI-988 2, and CVI-988 4, respectively). Agarose gel analysis of the samples from the real-time PCR for the samples obtained from the vaccinated birds suggested a DNA band of 60 bp (expected size for I1 amplicon). In the samples obtained from birds infected experimentally with RB-1B, only sample RB-1B 4 produced a signal during the assay (40-*C*t = 2.5). However, the reaction contained very little amount of specific DNA, as is shown in the agarose gel of Figure S1 panel B. 

### 3.7. Differential Expression of I1, I2, and I3 in RB-1B and CVI-988

At 5 days post-infection, all our three candidate introns (I1, I2, and I3) were differentially expressed in CEF cells infected with RB-1B and CVI-988. Such differential expression can be quantified using *GAPDH* as a PCR calibrator.

Briefly, to quantify precisely expression levels, standard curves using PCR-amplified or cloned fragments of DNA representing I1, I2, I3, and *GAPDH* were first made and used to compute PCR efficiency. The authenticity of PCR products was confirmed by Sanger sequencing. The expression levels of the three transcripts (I1, I2, I3) in CEF cells infected by RB-1B or CVI-988 at 5 days post-infection (*N* = 6) were computed and normalised against *GAPDH* in order to achieve accurate calibration. Fresh virus stock obtained after only two passages was used for infection for both of the viruses. Full details are described in the **Materials and Methods section**.

**Figure 7:**
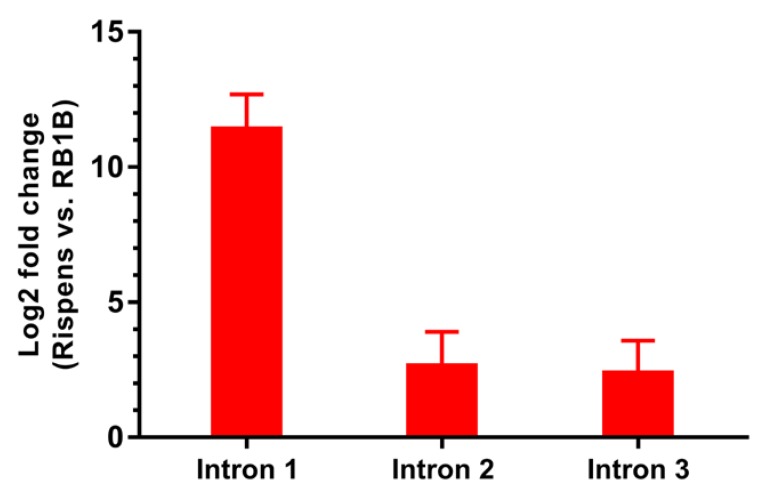
Differential expression in CEF cells of introns I1-I3 in CVI-988- and RB-1B-infected CEF cells. Different expression levels of introns I1, I2, and I3 when CEF cells were infected with 500 PFU of MDV strain CVI-988 were compared with cells infected with RB-1B at 5 days post-infection. The ratio of the expressions in CVI-988 and RB-1B was computed for each intron using the ΔΔ*C*T method and *GAPDH* as a calibrator (see the **Materials and Methods section**).

The results are presented in Figure 7 and represent one of the main outcomes of this paper. When comparing expression in CEF cells infected with CVI-988 to expression in CEF cells infected with RB-1B, the greatest fold change was observed for intron I1, which appeared to be expressed approximately 2800 times more when *GAPDH* was used as a calibrator. I2 and I3 exhibited fold changes that were lower than those of I1, but still easily measurable and statistically significant (I2 vs. *GAPDH*: 6.3; I3 vs. *GAPDH*: 5.3), confirming the ranking originally deduced from RNA-sequencing data (Table 3).

## 4. Discussion

The pathobiology of MDV is complex [44]. This alphaherpesvirus can infect chicken cells and cause tumours in a wide variety of tissues. The outcome is that virions are shed into the environment as infectious dander [6], whereas cell-free viruses can hardly be detected in vitro in the supernatants of any cell line. Some Mardivirus-specific genes have been identified and characterized, but more research is needed to understand the molecular mechanisms responsible for its complex life cycle, especially at the transcriptional level, unlike other herpesviruses whose transcriptomes have recently been studied, sometimes even in different tissues, such as *Herpes simplex virus*, *Pseudorabies virus*, *Epstein–Barr virus*, and *Cytomegalovirus* [12,13,15,27,28,45,46]. For a long time, an extensive characterisation of MDV transcripts has been lacking, with most of their characterisations being determined through Northern blot analysis and the nucleotide sequencing of a limited number of cDNA products, most notably the spliced variants of MDV oncogene *Meq* [30], *vIL8* [31], and *glycoprotein C* [33]. The recent study by Bertzbach and colleagues [47] provided more in-depth knowledge of MDV transcriptome in B cells. To expand on this, we reported on the viral transcriptome of MDV using high-throughput short-read RNA sequencing on RNA isolated from CEF cells infected with vvMDV strain RB-1B [48] and MDV vaccine strain CVI-988 [5].

Interestingly, and consistent with what can be observed across the *Herpesviridae* family, our results showed that RNA splicing was pervasive in CEF cells infected with these strains, giving rise to hundreds of so far unannotated spliceforms whose biological significance remain undefined and in need of further investigation. The complex transcription landscape that maps to the UL region of MDV suggests that in addition to transcripts encoding early and late gene products, other functional categories might be present, with a number of them possibly having regulatory functions in the control of gene products expression. This has been observed for both PSV and HSV1 [13,27,28]. For MDV in Bertzbach and colleagues’ study, the authors reported the I1 splicing isoform, which was studied in the present work. Interestingly, in the aforementioned study, the I1 splicing isoform was observed in B cells of chickens, which were inoculated with MDV strain CVI-988, but not in the B cells isolated from the MDV strain RB-1B-infected birds [47]. This is in line with our observation. In addition to the CEF infected cells, we could detect I1 in feather follicles of birds that had been vaccinated with MDV strain CVI-988 (Figure S1). In addition to I1, we observed other splicing isoforms that were lacking in the report by Bertzbach and colleagues—among them, the I2 and I3 isoforms. This could mean that I2 and I3 are specific for CEF cells. However, further studies are required to establish a firmer conclusion here.

One should note that there are limitations to the sensitivity of the technique employed—in particular, as explained in detail in the Materials and Methods section, spliceforms that are too weak with respect to the most abundant RNA species will not be detectable, depending on the number of sequencing reads generated during the experiment. As described in the Results section, several splicing isoforms identified in previous studies could not be found in our data. Whether this is due to limited sensitivity or to the fact that such isoforms are specific to other experimental conditions—for instance, [41] using MSB1 cells rather than CEF cells—the conclusion can only be that the potential size of the MDV transcriptome is even larger than what emerged from our study. Again, this is entirely similar to what can be seen for other herpesviruses—in previously studied CMV-infected cells, the authors were unable to identify some of the previously annotated genes [14].

Even more interestingly, a significant number of viral splicing isoforms of CVI-988 and RB-1B appeared to be strain-specific, despite the high genome sequence similarity (>99%) between the two strains. As far as we know, such a striking result has not been previously reported in the literature. Not surprisingly, our preliminary analysis of the host transcriptome indicated that the splicing of host transcripts may also depend on the virulence of the infecting strains. It is possible that the greater propensity for splicing exhibited by CVI-988 indirectly derives from the fact that its attenuation was obtained by repeated passaging the strain in cell culture, but by now the trait has apparently been fixed in its genome, which is an interesting biological fact. 

The discrepancy observed between the level of V.Ref measured from RNA-Seq data and the level of V.Ref measured from real-time PCR data could potentially be due to the BAC clone of the virus acting differently in cell culture compared to the virus itself. In addition, modifications during the generation of BAC clone could have impacted the virus in such a way that the virus did not replicate in exactly the same way as its wild type parental virus. However, it is unlikely that the observed discrepancy could have had an impact on our results. The expression level of I1 was normalised against the expression level of *GAPDH* (a cellular marker). Normalising the expression level of I1 against V.Ref yielded an even larger difference in the expression level of I1 between the two strains.

Taken together, these results suggest the possibility that different MDV strains might interact differently with the splicing machinery of the host; a different interaction of the spliceosome and associated proteins with the MDV genome in CEF cells infected by CVI-988 might result in a much richer splicing landscape. However, it is not possible at this stage to pinpoint the responsible factor for such differences at a molecular level. For instance, in the *ICP27* region, our data showed similar splicing patterns for RB-1B and CVI-988 (with only one intron present in CVI-988 and not in RB-1B), and no differential expression (corrected *p*-value for differential expression computed by edgeR = 0.65), and thus it is unclear at the moment whether *ICP27* might be responsible for what we observed. Different splice isoforms of the major MDV oncogene *Meq* have been reported (25), and a recent study has demonstrated functional differences in pathogenicity of viruses expressing different splice forms derived from CVI-988 [49]. Although only one *Meq* isoform was identified in our study, the isoforms of viral proteins, which may differ between viral strains, appear to be important. At any rate, further work is required to investigate a possible relation between the reported splice isoforms and virus pathogenicity—their significance and relation with the known virus pathogenesis factors are presently unknown.

As the results of RNA-sequencing describe the averaged collective nature of a relatively large number of infected cells, it is hard to say whether these differences are typical of every infected cell or for just a smaller sub-population. To truly reveal the complexity of alternative splicing across diverse cell and tissue types, in future studies, it might be prudent to sequence the RNA transcriptome of individual cells, especially from tissues (lymphocytes, tumours, FFE, etc.) of experimentally infected birds, and use proteomics to determine whether splice variant transcripts are translated into protein products or only have RNA regulatory functions. We also plan to define the virus transcriptome in various tissues isolated from vaccinated/challenged birds that are susceptible and resistant to Marek’s disease. That might help elucidate the molecular mechanisms underlying the diversity that we observed in splicing landscapes.

Overall, the data presented in this report suggest a remarkable amount of spliced viral transcripts in infected CEF cells, allowing us to identify spliced transcripts that contain introns exclusive to the vaccine strain CVI-988. Building upon such findings, we also designed primers and probes that can specifically detect such transcripts, thus effectively differentiating the transcriptome of MDV strain CVI-988 from that of strain RB-1B in CEF cells. To the best of our knowledge, this study is the first to ever propose a technique based on the differential detection of splicing events. Obvious questions to be elucidated in the future are whether our results can be extended to other tissues/MDV strains, and whether differential expression of I1 (or possibly other spliceforms yet to be identified) can also be detected in vivo in a larger study.

## Figures and Tables

**Table 1:** Alignment statistics for our dataset (reads were produced using a directional sequencing protocol). To each read, one or more alignments can correspond (for instance, a read might align to both terminal repeats).

MDV Strain	Reads Mapping to the Forward Viral Strand	Alignments to the Forward Viral Strand	Reads Mapping to the Reverse Viral Strand	Alignments to the Reverse Viral Strand
CVI-988 replicate 1	949,845	1,273,630	639,578	966,599
CVI-988 replicate 2	1,391,374	1,865,743	932,231	1,404,683
RB-1B replicate 1	717,633	1,213,552	549,656	998,707
RB-1B replicate 2	650,431	1,110,938	496,594	914,609

**Table 2:** Enumeration of putative novel coding splicing isoforms for several MDV genes, as deduced from introns observed in CEF cells infected by MDV strains RB-1B and CVI-988.

Genomic Region	Spliceforms In CVI-988	Spliceforms In RB-1B	Function
*vIL8*	5	5	Viral IL8
*pp14A/B*	6	5	14KD lytic proteins
*Lip*	3	2	Lipase
*UL3*	5	3	Nuclear phosphoprotein
*UL15*	17	4	DNA packaging protein
*UL19*	4	4	Major capsid protein
*UL21*	3	2	Tegument protein
*UL24*	2	2	Reactivating from latency/immune evasion
*UL28*	7	1	DNA packaging protein.
*UL29*	2	1	DNA binding protein
*UL34*	2	1	Membrane phosphoprotein
*UL38*	2	1	Capsid protein
*UL41*	4	1	Tegument protein
*UL44*	6	5	Glycoprotein C
*UL46*	2	1	Tegument phosphoprotein
*UL47*	11	1	Tegument phosphoprotein
*UL48*	3	3	Tegument immediate-early protein
*UL49*	2	2	Tegument phosphoprotein
*UL52/UL53/UL54*	14	4	DNA helicase/glycoprotein *K/ICP27*-like proteins
*RLORF14a*	3	3	38 KD protein
*ICP4*	5	1	IE protein
*ICP4* area	15	7	-
*US7*	5	3	Glycoprotein I
*US8*	3	3	Glycoprotein E

**Table 3:** List of introns that are (A) spliced exclusively in MDV strain CVI-988, or (B) spliced exclusively in MDV strain RB-1B. (C) The intron, which was chosen as the viral reference (V.Ref) in *ICP4/LAT*. To make splicing events occurring in different MDV strains comparable, all introns are listed in terms of coordinates on the basis of the genomic sequence of MDV reference strain MD5/GaHV2 (NCBI accession NC_002229.3). Mapping the introns occurring in the RB-1B/CVI-988 transcriptomes to the MD5 genome was possible thanks to the extremely high sequence similarity (>99%) shared by MD5, RB-1B, and CVI-988. A full table with intron coordinates to all the three strains is provided in Table S2.

MDV Strain	Strand	Position of First Intron Nucleotide	Position of Last Intron Nucleotide	Gene	Read Coverage	Name
A. Introns only spliced in MDV strain CVI-988						
GaHV2	−	112359	111959	*VP22* gene, intergenic region	646	I1
GaHV2	−	112881	112500	*UL50*	58	I2
GaHV2	+	43021	43723	*UL15*, terminase	39	I3
GaHV2	+	43021	43166	*UL15*, terminase	42	-
GaHV2	+	50934	51306	*UL21*	38	
GaHV2	−	108334	107252	*UL46/UL47*	29	
GaHV2	+	43021	43808	*UL15*, terminase	28	
B. Introns only spliced in MDV strain RB-1B						
GaHV2	−	111959	112277	*UL49/UL49.5*	19	-
C. The intron spliced in cells infected with both of the strains						
GaHV2	−	170198	170114	*ICP4/LAT*	183	V.Ref

## Supplementary Materials

The following are available online at https://www.mdpi.com/1999-4915/12/3/329/s1: **Table S1**: List of introns that are spliced in MDV strains CVI-988 and RB-1B; **Table S2**: List of introns that are spliced exclusively in MDV strain CVI-988, strain RB-1B, or equally spliced in the two strains; **Table S3**: PCR primers and probles; **Figure S1**: Detection of I1 in feather samples.
