# Supplementary material for: Pervasive Differential Splicing in Marek’s Disease Virus Can Discriminate CVI-988 Vaccine Strain from RB-1B Very Virulent Strain in Chicken Embryonic Fibroblasts
Source: Viruses. 2020 Mar 18;12(3):329. doi: 10.3390/v12030329 (PMC7150913; doi:10.3390/v12030329)

Pervasive Differential Splicing in Marek's Disease Virus can Discriminate CVI-988 Vaccine Strain from RB-1B Very Virulent Strain in Chicken Embryonic Fibroblasts

Supplementary materials

Yashar Sadigh [1,†], Abdessamad Tahiri-Alaoui [2,†], Stephen Spatz [3], Venugopal Nair [1,*] and Paolo Ribeca [4,5,*]

[1] Avian Viral Oncogenesis, The Pirbright Institute, Ash Road, Woking GU24 0NF, UK; yashar.sadigh@pirbright.ac.uk
[2] Clinical BioManufacturing Facility, The Jenner Institute, University of Oxford, Old Road, Headington, Oxford OX3 7JT, UK; abdessamad.tahiri-alaoui@ndm.ox.ac.uk
[3] US National Poultry Research Center, 934 College Station Road, Athens, GA 30605, USA; stephen.spatz@ars.usda.gov
[4] Integrative Biology and Bioinformatics, The Pirbright Institute, Ash Road, Woking GU24 0NF, UK;
[5] Biomathematics and Statistics Scotland (BioSS), James Clerk Maxwell Building, Peter Guthrie Tait Road, The King's Buildings, Edinburgh EH9 3FD, UK;
* Correspondence: venugopal.nair@pirbright.ac.uk (V.N.); paolo.ribeca@bioss.ac.uk (P.R.); Tel.: +44 1483232441 (V.N.), +44 1316507547 (P.R.)
† These authors contributed equally to this work.

Table S1. List of introns that are: (**Panel A**) spliced in MDV strain CVI-988; (**Panel B**) spliced in MDV strain RB-1B – after filtering out introns with low-coverage (<10 reads) and introns which are not spliced in all biological replicates. To make splicing events occurring in different MDV strains comparable, all introns are listed in terms of coordinates based on the genomic sequence of MDV reference strain MD5 (NCBI accession NC_002229.3). Mapping the introns occurring in the RB-1B/CVI-988 transcriptomes to the MD5 genome was possible thanks to the extremely high sequence similarity (>99%) shared by MD5, RB-1B and CVI-988.

MDV strain	Strand	Position of first intron nucleotide	Position of last intron nucleotide	Read coverage	MDV strain	Strand	Position of first intron nucleotide	Position of last intron nucleotide	Read coverage
**A. Introns spliced in MDV strain CVI-988**					**B. Introns spliced in MDV strain RB-1B**				
MD5	+	103285	103388	9435	MD5	+	138129	138303	4052
MD5	+	17540	17621	4753	MD5	-	3317	3491	3974
MD5	+	138129	138303	4415	MD5	-	3083	3180	3378
MD5	-	3317	3491	4325	MD5	+	138440	138537	3366
MD5	+	103244	103388	4145	MD5	+	128516	130900	3113
MD5	-	3083	3180	3994	MD5	-	10720	13104	3105
MD5	+	138440	138537	3972	MD5	+	17540	17621	2043
MD5	+	128516	130900	3666	MD5	+	103285	103388	1816
MD5	-	10720	13104	3663	MD5	+	129866	130900	1013
MD5	-	127855	128016	1893	MD5	-	10720	11754	1006
MD5	+	13604	13765	1801	MD5	-	127855	128016	972
MD5	-	126904	127407	1236	MD5	+	13604	13765	953
MD5	+	129866	130900	879	MD5	+	103244	103388	917
MD5	-	10720	11754	876	MD5	-	10720	13048	825

MD5	+	14631	14700	778	MD5	+	128572	130900	814
MD5	-	126904	128016	659	MD5	-	126904	127407	588
MD5	-	111959	112359	646	MD5	-	10720	12856	571
MD5	-	164391	164465	596	MD5	+	128764	130900	564
MD5	+	56222	57133	547	MD5	-	164391	164465	533
MD5	-	10720	12856	516	MD5	+	14631	14700	409
MD5	+	128764	130900	508	MD5	+	13604	14477	230
MD5	-	10720	13048	489	MD5	-	126904	128016	219
MD5	+	128572	130900	481	MD5	-	170766	170836	219
MD5	+	14631	17621	481	MD5	+	147608	147678	213
MD5	+	13604	14477	394	MD5	-	127939	128016	202
MD5	+	23570	25010	388	MD5	-	170114	170198	191
MD5	+	130693	130900	361	MD5	+	13604	13681	188
MD5	-	10720	10927	351	MD5	+	23570	25010	186
MD5	-	170766	170836	293	MD5	-	170288	170657	147
MD5	+	147608	147678	289	MD5	+	147123	147209	138
MD5	-	127939	128016	279	MD5	-	164391	170037	138
MD5	+	43966	44225	270	MD5	+	148246	148330	137
MD5	+	13604	13681	261	MD5	+	147787	148156	132
MD5	-	170114	170198	260	MD5	-	171235	171321	127
MD5	-	130958	131423	245	MD5	-	127917	128016	127
MD5	+	48633	48904	245	MD5	-	3099	3180	122
MD5	+	42651	42884	237	MD5	+	138440	138521	121
MD5	+	10197	10662	232	MD5	+	130693	130900	118
MD5	+	49048	50816	221	MD5	-	10720	10927	117
MD5	-	170288	170657	203	MD5	+	13604	13703	116
MD5	-	127917	128016	201	MD5	+	42651	42884	102
MD5	+	117624	118995	194	MD5	+	56222	57133	97
MD5	-	164391	170037	192	MD5	+	39404	42884	91
MD5	+	148246	148330	192	MD5	-	130958	131423	89
MD5	+	10755	11811	191	MD5	+	10197	10662	83
MD5	+	147787	148156	190	MD5	+	14631	17621	81
MD5	-	129809	130865	187	MD5	-	126269	128016	70
MD5	+	13604	13703	179	MD5	+	14218	14477	66
MD5	+	118203	118995	168	MD5	-	129809	130865	64
MD5	+	44447	46036	166	MD5	+	13602	13765	62
MD5	-	150945	152141	163	MD5	+	117624	118995	62
MD5	+	166303	167499	162	MD5	+	162270	162416	61
MD5	+	147123	147209	158	MD5	-	126904	127437	60
MD5	+	14218	14477	153	MD5	-	127855	128018	60
MD5	-	171235	171321	151	MD5	+	10755	11811	60
MD5	+	162270	162416	148	MD5	-	170772	170836	55
MD5	-	3099	3180	143	MD5	-	48026	48551	55
MD5	+	138440	138521	141	MD5	+	147608	147672	53
MD5	+	43444	43723	136	MD5	+	141646	147059	52
MD5	-	126269	128016	130	MD5	-	171385	176798	51
MD5	+	39404	42884	121	MD5	+	144724	144996	51
MD5	+	103562	104515	120	MD5	-	10720	14783	50

MD5	+	17406	17621	116	MD5	-	173448	173720	48
MD5	-	108478	109362	115	MD5	-	164391	170836	48
MD5	-	110865	111173	111	MD5	+	48633	48904	47
MD5	+	13602	13765	109	MD5	-	131633	131762	45
MD5	-	127855	128018	102	MD5	+	145239	147059	44
MD5	+	9858	9987	101	MD5	+	9858	9987	44
MD5	-	131633	131762	101	MD5	+	13604	17621	44
MD5	+	100540	103388	97	MD5	-	171385	173205	43
MD5	-	10720	14783	97	MD5	+	128516	129736	43
MD5	+	13604	14700	96	MD5	-	11884	13104	42
MD5	-	48026	48551	94	MD5	+	100540	103388	41
MD5	+	50934	55652	81	MD5	+	166303	167499	41
MD5	+	37541	42884	81	MD5	-	150945	152141	40
MD5	-	126904	128018	71	MD5	+	128572	129736	37
MD5	-	171385	173205	69	MD5	+	118203	118995	37
MD5	+	145239	147059	68	MD5	+	43966	44225	36
MD5	+	43444	43808	67	MD5	+	13602	14477	35
MD5	-	170772	170836	66	MD5	-	11884	13048	34
MD5	+	147608	147672	64	MD5	+	13604	14700	34
MD5	+	144724	144996	62	MD5	-	164391	170657	34
MD5	+	161471	162416	60	MD5	-	156473	156727	32
MD5	+	14631	14710	60	MD5	-	164391	166164	31
MD5	-	173448	173720	59	MD5	-	174496	175095	31
MD5	-	112500	112881	58	MD5	-	135161	135780	30
MD5	+	5840	6459	57	MD5	+	5840	6459	30
MD5	-	63796	64600	56	MD5	-	170625	170836	30
MD5	-	135161	135780	55	MD5	+	143349	143948	29
MD5	-	110697	111173	55	MD5	+	147608	147819	29
MD5	-	164391	170836	53	MD5	+	148407	150128	29
MD5	+	138002	138303	52	MD5	-	168316	170037	28
MD5	-	165820	166164	51	MD5	-	126899	127407	27
MD5	-	126899	127407	50	MD5	-	124414	124816	26
MD5	-	124414	124816	49	MD5	-	110865	111173	26
MD5	+	152280	152624	48	MD5	-	164391	176798	25
MD5	+	161498	162416	48	MD5	+	161471	162416	25
MD5	+	159632	162416	48	MD5	-	126904	128018	24
MD5	+	49048	49142	48	MD5	+	43444	43723	24
MD5	-	10720	12337	48	MD5	+	17406	17621	24
MD5	+	129283	130900	47	MD5	-	11884	12856	23
MD5	-	156473	156727	47	MD5	+	103562	104515	23
MD5	+	50934	56108	45	MD5	+	128764	129736	23
MD5	+	5558	5677	43	MD5	+	138002	138303	22
MD5	-	135943	136062	43	MD5	-	126269	127407	20
MD5	+	43021	43166	43	MD5	+	21286	21379	20
MD5	+	13604	17621	42	MD5	-	111959	112277	19
MD5	+	115207	118995	42	MD5	+	49048	50816	19
MD5	-	3317	3618	42	MD5	-	168978	170037	19
MD5	-	143380	152141	41	MD5	-	164391	168893	18

MD5	-	164391	170657	41
MD5	+	166303	175064	40
MD5	+	42798	42884	40
MD5	+	43021	43723	39
MD5	+	37541	39215	38
MD5	+	50934	51306	38
MD5	+	21286	21379	38
MD5	+	120317	120767	38
MD5	+	13602	14477	37
MD5	+	46711	47839	34
MD5	-	72063	72993	34
MD5	-	126269	127407	33
MD5	+	44447	48559	32
MD5	-	107252	108334	31
MD5	+	21581	25010	31
MD5	+	13608	13765	31
MD5	+	46711	48559	30
MD5	-	107030	109362	30
MD5	-	166272	170037	30
MD5	-	127855	128012	30
MD5	+	168066	169768	29
MD5	-	148676	150378	29
MD5	-	13584	14783	29
MD5	+	148407	152172	28
MD5	+	135165	138303	28
MD5	-	3317	6455	28
MD5	+	16981	17621	27
MD5	-	10720	12469	26
MD5	-	73286	73548	26
MD5	+	129151	130900	25
MD5	+	126410	126729	25
MD5	+	120343	120767	25
MD5	-	174496	175095	25
MD5	+	143349	143948	25
MD5	+	43021	43808	25
MD5	-	164391	170198	24
MD5	-	126983	127407	23
MD5	-	122846	124039	23
MD5	+	137675	138303	23
MD5	-	3317	3945	23
MD5	+	56222	65913	23
MD5	-	164391	170046	22
MD5	-	150744	152141	22
MD5	+	144052	144376	22
MD5	+	166303	167700	21
MD5	-	168316	170037	21
MD5	+	126300	126368	21
MD5	-	46921	47956	21

MD5	+	148407	149466	18
MD5	-	165820	166164	18
MD5	-	127855	128012	18
MD5	+	137675	138303	17
MD5	+	14631	14710	17
MD5	+	129283	130900	17
MD5	-	10720	12337	17
MD5	-	3317	3945	16
MD5	-	170288	170670	16
MD5	+	17521	17621	16
MD5	-	131683	131762	16
MD5	+	42798	42884	16
MD5	+	152280	152624	15
MD5	+	161498	162416	15
MD5	+	13608	13765	15
MD5	+	37541	42884	15
MD5	+	149551	149915	14
MD5	-	3317	3618	14
MD5	+	49048	49142	13
MD5	-	168529	168893	13
MD5	+	5558	5677	13
MD5	-	135943	136062	13
MD5	-	10720	12851	13
MD5	+	148407	152172	12
MD5	+	168066	169768	12
MD5	-	126983	127407	12
MD5	-	164391	170046	12
MD5	+	147774	148156	12
MD5	+	21290	21379	11
MD5	+	129151	130900	11
MD5	+	126858	126943	11
MD5	-	143380	152141	11
MD5	+	166303	175064	11
MD5	+	144052	144604	11
MD5	-	173840	174392	11
MD5	-	10720	12469	11
MD5	-	46921	47956	11
MD5	-	65190	65574	10
MD5	+	144052	144376	10
MD5	-	174068	174392	10
MD5	+	130045	130114	10
MD5	-	11506	11575	10
MD5	+	115207	118995	10
MD5	+	79750	103388	10

MD5	-	174068	174392	21
MD5	+	148407	150128	20
MD5	-	147498	152141	20
MD5	-	107243	108334	20
MD5	-	47990	48551	20
MD5	+	166303	170946	20
MD5	-	14872	15396	19
MD5	+	5840	6671	19
MD5	-	170288	170670	19
MD5	+	37665	42884	19
MD5	-	97915	98336	19
MD5	-	63796	64124	18
MD5	+	49665	50816	18
MD5	+	144850	144996	18
MD5	-	63796	64641	18
MD5	+	21290	21379	18
MD5	+	166303	170579	17
MD5	+	13602	13703	17
MD5	+	46711	48493	17
MD5	+	65352	65913	17
MD5	-	147865	152141	17
MD5	+	161471	162075	17
MD5	-	132126	133050	17
MD5	+	133179	134823	17
MD5	+	39404	42283	16
MD5	+	129866	130599	16
MD5	+	114816	118995	16
MD5	-	69904	72062	16
MD5	-	3317	4065	16
MD5	+	137555	138303	16
MD5	+	49048	50820	16
MD5	+	147774	148156	16
MD5	-	170625	170836	16
MD5	-	6797	8441	16
MD5	-	131683	131762	16
MD5	+	9858	9937	16
MD5	+	65264	65913	16
MD5	+	158277	162416	16
MD5	+	126858	127378	16
MD5	+	147608	147819	16
MD5	+	43021	55652	15
MD5	-	15548	16661	15
MD5	-	126269	128018	15
MD5	+	17521	17621	15
MD5	+	47947	48559	15
MD5	+	43021	44225	15
MD5	+	9854	9987	14
MD5	+	146193	147059	14

MD5	+	130045	130114	14
MD5	-	11506	11575	14
MD5	-	126904	127060	14
MD5	-	32229	32414	14
MD5	-	107568	108334	14
MD5	+	144494	144604	14
MD5	+	44447	48493	14
MD5	-	124414	125781	14
MD5	+	43021	56108	14
MD5	+	130778	130900	13
MD5	-	173840	173950	13
MD5	-	155921	156727	13
MD5	-	147498	150378	13
MD5	+	168066	170946	13
MD5	-	3083	3176	13
MD5	+	13602	14700	13
MD5	+	128516	129736	13
MD5	+	44447	48904	13
MD5	+	43282	43389	13
MD5	+	44447	45636	12
MD5	+	50934	65913	12
MD5	+	138444	138537	12
MD5	-	53654	54762	12
MD5	-	171385	172251	12
MD5	+	79750	91658	12
MD5	-	171385	176798	11
MD5	+	11901	13106	11
MD5	-	128514	129719	11
MD5	-	173840	174392	11
MD5	+	144052	144604	11
MD5	+	114627	118995	11
MD5	+	58262	59074	10
MD5	+	4834	5677	10
MD5	-	111487	112359	10
MD5	+	141646	147059	10
MD5	+	126858	126943	10
MD5	+	113029	113551	10
MD5	-	107042	109362	10
MD5	+	132975	133061	10
MD5	-	111959	112157	10

Table S2. List of introns that are: (**Panel A**) spliced exclusively in MDV strain CVI-988; (**Panel B**) spliced exclusively in MDV strain RB-1B; (**Panel C**) equally spliced in MDV strains RB-1B and CVI-988. In order to make splicing events occurring in different MDV strains comparable, all introns are listed in terms of coordinates based on the genomic sequence of MDV reference strain MD5 (NCBI accession NC_002229.3), CVI-988 (accession number DQ530348) and RB-1B (accession number EF523390). Identifying introns in MD5, RB-1B and CVI-988 was possible thanks to the extremely high sequence similarity (>99%) shared by the three strains. Read coverages can exhibit small variations due to minor local sequence differences.

MDV strain	Strand	Position of first intron nucleotide	Position of last intron nucleotide	Read coverage	Gene	Name
**A. Introns only spliced in MDV strain CVI-988**						
MD5	-	112359	111959	646	UL49/UL49.5	I1
RB-1B		112673	113073	646		
CVI-988		112335	112735	643		
MD5	-	112881	112500	58	UL49.5	I2
RB-1B		113214	113595	58		
CVI-988		112876	113257	58		
MD5	+	43021	43723	39	UL15	I3
RB-1B		43689	44391	39		
CVI-988		43469	44171	39		
MD5	+	43021	43166	42	UL15	-
RB-1B		43689	43834	43		
CVI-988		43469	43614	43		
MD5	-	108334	107252	29	UL46/UL47	-
RB-1B		107966	109048	31		
CVI-988		107646	108728	31		
MD5	+	43021	43808	28	UL15	-
RB-1B		43689	44476	25		
CVI-988		43469	44256	25		
MD5	+	50934	51306	38	UL21	-
RB-1B		51602	51974	38		
CVI-988		51382	51754	38		
**B. Introns only spliced in MDV strain RB-1B**						
MD5	-	111959	112277	19	UL49/49.5	-
RB-1B		112673	112991	19		
CVI-988		112335	112653	19		
**C. Intron being equally spliced in MDV strains RB-1B and CVI-988**						
MD5	-	170114	170198	260	LAT	V.REF
RB-1B		170838	170922	262		
CVI-988		171192	17126	262		

Table S3. PCR primers and probes used in the paper. They correspond to introns I1, I2, I3 and V.REF of **Table S2**; an additional probe was designed to target an intron of the *GAPDH* gene (see **Materials and Methods section**).

Name	Primer F	Primer R	Probe
I1	ACGCCGTTCCGATTTCCGCC	AAATCACGATCTCGACGAGGATGG	TCCCCCATGATAATGCATTATGAATGTCCAT (FAM/ZEN/3IABLFQ)
I2	CTGCAATTCCGTGTCGATGA	CTGACAGCAGTTTCTATCGAAGT	AAACGGTTGGACCTAGAATGTGGTCC (FAM/ZEN/3IABLFQ)
I3	GGTTTTTGAATCAAGCTACTTGCA	CTTCTCATTGCCCCATCCAT	ACAGTGCCTGTTTTCAT (FAM/ZEN/3IABLFQ)
GAPDH	TGGGTGTCAACCATGAGAAATA	ACCCTCCACAATGCCAAA	ATTGTCAGCAATGCATCGTGCACC (FAM/ZEN/3IABLFQ)
V.REF	AATCCCTGGACCGGTTTG	GAGGCAGCGTGGTAGAT	CGCAGCCAGGCATTTAAAGTCTTTCC (FAM/ZEN/3IABLFQ)

Figure S1. Detection of I1 in feather samples. **Panel A** shows 40-Ct values from feather samples experimentally infected with MDV strain RB-1B or vaccinated with MDV strain CVI-988 (*N* = 4; a no template control labelled NTC is also present). In **Panel B**, the 2% agarose gel corresponding to the PCR reactions shown in **panel A** is displayed. A 60 base-pair band (location shown by the arrow on the gel) is detected with I1-specific primers.

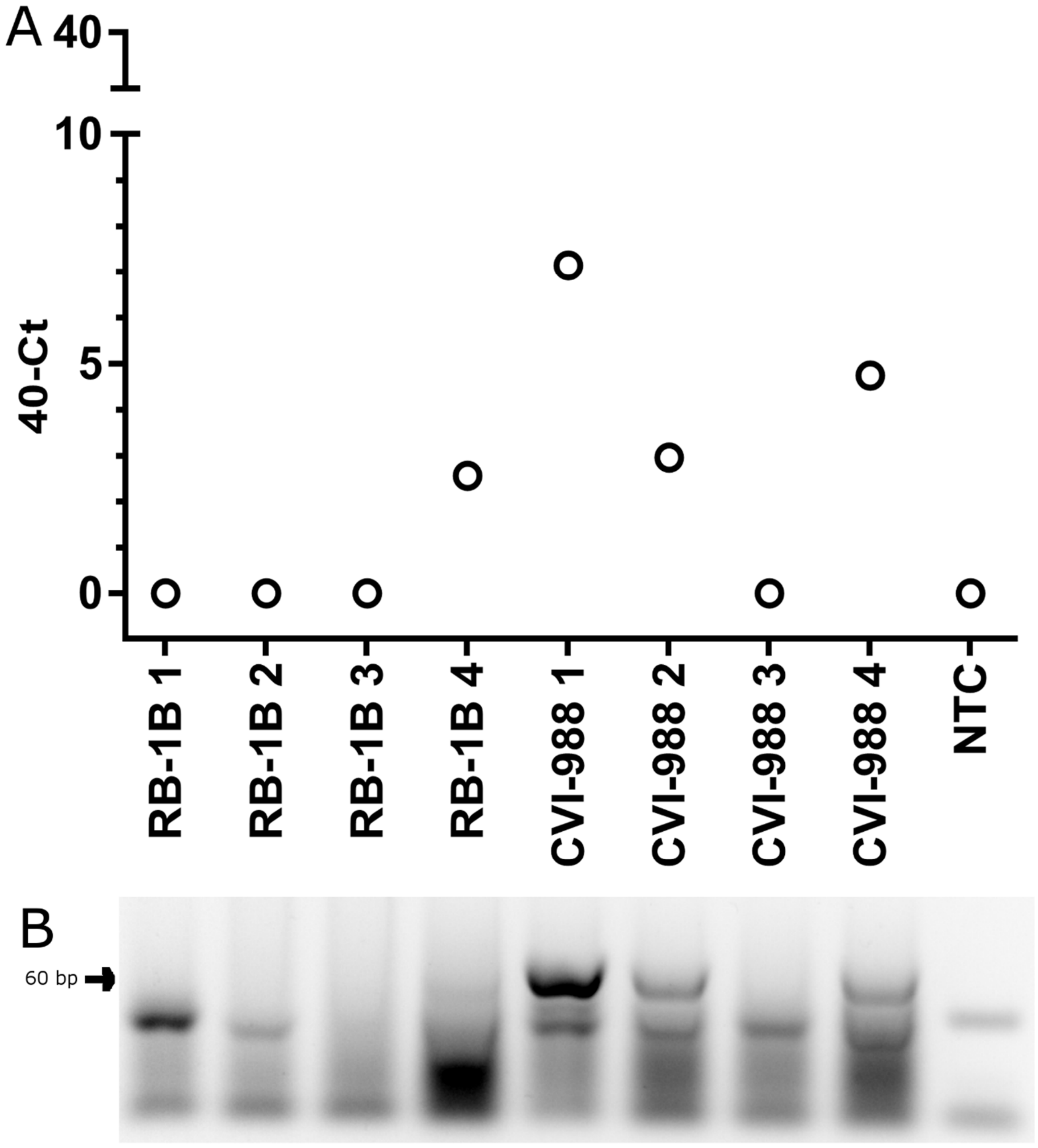

Supplement: Supplementary file 1 [file viruses-12-00329-s001.pdf]
